# Spatial Beam Self-Cleaning in Second-Harmonic Generation

**DOI:** 10.1038/s41598-020-64080-7

**Published:** 2020-04-29

**Authors:** K. Krupa, R. Fona, A. Tonello, A. Labruyère, B. M. Shalaby, S. Wabnitz, F. Baronio, A. B. Aceves, G. Millot, V. Couderc

**Affiliations:** 1 Institute of Physical Chemistry of the Polish Academy of Sciences, ul. Kasprzaka 44/52, 01-224 Warsaw, Poland; 2 Université Bourgogne Franche-Comté, ICB, UMR CNRS 6303, 9 Av. A. Savary, 21078 Dijon, France; 3 Dipartimento di Ingegneria dell’Informazione, Università di Brescia, Via Branze 38, 25123 Brescia, Italy; 4 Université de Limoges, XLIM, UMR CNRS 7252, 123 Av. A. Thomas, 87060 Limoges, France; 5 DIET, Sapienza University of Rome, Via Eudossiana 18, 00184 Rome, Italy; 6 Novosibirsk State University, Pirogova 1, Novosibirsk, 630090 Russia; 7 Department of Mathematics, Southern Methodist University Dallas, Texas, 75275-0156 USA; 8 Physics Department, Faculty of Science, Tanta University, 31527 Tanta, Egypt; 9 Zerogroup, Brescia, Italy

**Keywords:** Nonlinear optics, Nonlinear optics

## Abstract

We experimentally demonstrate the spatial self-cleaning of a highly multimode optical beam, in the process of second-harmonic generation in a quadratic nonlinear potassium titanyl phosphate crystal. As the beam energy grows larger, the output beam from the crystal evolves from a highly speckled intensity pattern into a single, bell-shaped spot, sitting on a low energy background. We demonstrate that quadratic beam cleanup is accompanied by significant self-focusing of the fundamental beam, for both positive and negative signs of the linear phase mismatch close to the phase-matching condition.

## Introduction

There is currently an intense research interest in nonlinear multimode optical systems. In the case of longitudinal (or temporal frequency) modes, nonlinear optical micro resonators permit to couple a single longitudinal laser mode to a large number of microcomb modes^1^. Conversely, in the case of transverse (or spatial frequency) modes, third-order nonlinear optical processes have been exploited to convert a highly multimode optical beam into a quasi-singlemode beam^2^. Important examples include the process of stimulated Brillouin^3^ and Raman scattering^4,5^, respectively, in graded-index (GRIN) multimode optical fibers (MMFs), whereby a highly multimode pump beam generates a frequency down-converted, high quality Stokes beam carried by the fundamental mode of the fiber. The nonlinear scattering induced recovery of the beam quality, or nonlinear beam cleanup, has enabled significant applications such as coherent beam combining^6^, and the development of high-power, high beam quality fiber amplifiers^7^ and lasers based on MMFs^8,9^.

Not until quite recently, it has been observed that a high-power beam propagating in GRIN MMFs may undergo a self-induced spatial cleaning via the intensity dependent contribution to the refractive index, or Kerr effect^10–13^. Spatial beam self-cleaning in MMFs has the potential to be exploited for high power laser beam delivery applications, as a fast saturable absorber mechanism for high-peak power fiber laser mode-locking^14–17^, for the energy up-scaling of fiber supercontinuum light sources^18,19^, and for increasing the performance of multiphoton microscopy and endoscopy techniques^20^. Kerr beam self-cleaning is characterized by a redistribution of energy among the initially excited fiber modes, while the average mode number remains invariant along the propagation, even in the highly nonlinear regime^21^. Nonlinearity leads to chaotic nonlinear wave mixing: as a result, a flow of energy towards the fundamental mode of the fiber, or condensate, is accompanied by a simultaneous flow of energy into higher-order modes^22,23^. This phenomenology is analogous to the process of 2D turbulence in hydrodynamics, where a direct cascade towards high wave numbers is accompanied by an inverse cascade into the condensate^21^.

The condensation of electromagnetic waves upon propagation in a nonlinear medium is a general physical process, which originates from the natural thermalization of conservative Hamiltonian wave systems, leading to an irreversible evolution toward the thermodynamic equilibrium state which maximizes entropy^22–25^. The formation of a condensate can also be seen as the emergence of a universal statistical attractor, resulting from soliton turbulence in a non-integrable wave system^26^. Thus, classical wave condensation is not limited to the case of a pure cubic Kerr nonlinearity: it has been numerically predicted to occur in media with either a nonlocal or with a saturable cubic nonlinearity^27^, as well as in the parametric wave mixing occurring in quadratic materials^28,29^. In their experiments, Sun *et al*.^30^ clearly demonstrated wave condensation of an initially spatially chaotic beam into the fundamental plane wave mode, with k = 0, in a SBN:75 (Sr0:75Ba0:25Nb2O6) photorefractive crystal, exhibiting a self-defocusing (repulsive) nonlinearity, controlled by applying a voltage across the c axis of the crystal.

In this work, we experimentally demonstrate a novel mechanism for nonlinear spatial self-cleaning of a highly multimode (i.e., comprising a large number of spatial frequency components) optical beam into the plane wave mode or component, based on the process of second-harmonic generation (SHG) in a quadratic nonlinear optical crystal. The generation of the SH of a ruby laser in crystalline quartz was the first nonlinear optical experiment, reported by Franken *et al*. as early as 1961^31^. Since then, SHG has grown to be the most established nonlinear optical effect, with widespread use across laser technologies. Nevertheless, the potential of SHG for spatial beam self-cleaning has so far not been fully appreciated.

The existence of nonlinear self-sustained beams and associated beam reshaping at negative mismatch values was earlier demonstrated for quadratic spatial solitons^32,33^, and, more recently, for polychromatic filaments^34^. Surprisingly, here we show that nonlinear beam cleanup may occur for initially fully speckled beams, albeit in a limited region around phase-matching, for both signs of the linear phase mismatch.

In order to show that, we employed a coherent, quasi-continuous wave (CW) laser beam, spatially scrambled by propagation in a short segment of highly multimode optical fiber rod. Next, this beam was coupled to a quadratic nonlinear potassium titanyl phosphate (KTP) bulk crystal. Whenever the phase-matching for SHG is exactly (or nearly) satisfied, and the laser beam energy is sufficiently high, we observed that the output beam undergoes a spontaneous recovery of its spatial quality.

Numerical simulations, based on the general model of three-wave mixing in bulk media, have been performed, and show a good qualitative agreement with our observations. Close to phase-matching of the SHG process, propagation of the FF beam can be described in terms of an equivalent third-order non-local nonlinear response^35^ (see the Supplementary for details). This permits us to conjecture a possible connection between the mechanism of self-cleaning in instantaneous Kerr media (*e.g*., multimode optical fibers), and beam self-cleaning in SHG. Indeed, our results provide a new example of nonlinear field coalescence in nonlocal nonlinear media. This effect was theoretically predicted by Conti *et al*. in the nonlinear quadratic framework^36^, and later experimentally confirmed in terms of spectral cooling upon nonlinear propagation in a bulk liquid crystal^37^. However, future work, with the aim of developing a general understanding of the complex nonlinear dynamics of multimode wave systems, is required.

We further analyse the mechanism of SHG-induced beam cleaning by characterizing, in the strong conversion regime around the phase-matching condition, the nonlinear spatial response of the crystal with a quasi-plane wave beam. We show that the SH conversion efficiency broadens with fundamental frequency (FF) power, in agreement with a simple plane-wave model. Moreover, our experiments reveal that the FF beam experiences spatial compression or self-focusing for both positive and negative wave vector mismatch values. In addition, FF beam cleaning occurs in a regime where the SHG efficiency is strongly reduced by nonlinear saturation effects, owing to the interplay of beam self-focusing and walk-off^38^.

## Results

### Spatial beam self-cleaning

**Figure 1:**
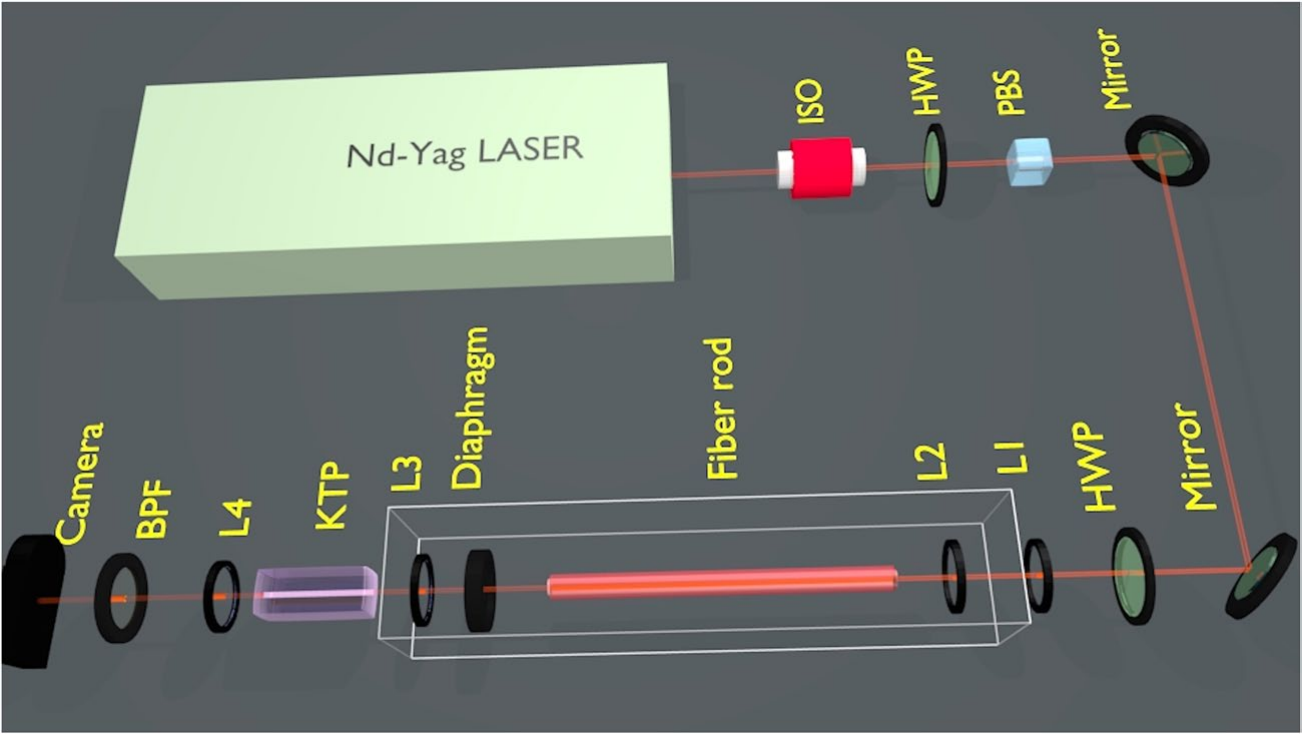
Experimental setup. To evaluate the nonlinear spectral acceptance, the laser beam is directly injected in the crystal. In the beam cleaning experiments discussed in section 4, the speckled beam is obtained by the insertion of a multimode optical fiber rod (white box). L1, L2, L3, L4: lenses; HWP: half-wave plate; BPF: bandpass filter; ISO: isolator; PBS: polarisation beam splitter.

**Figure 2:**
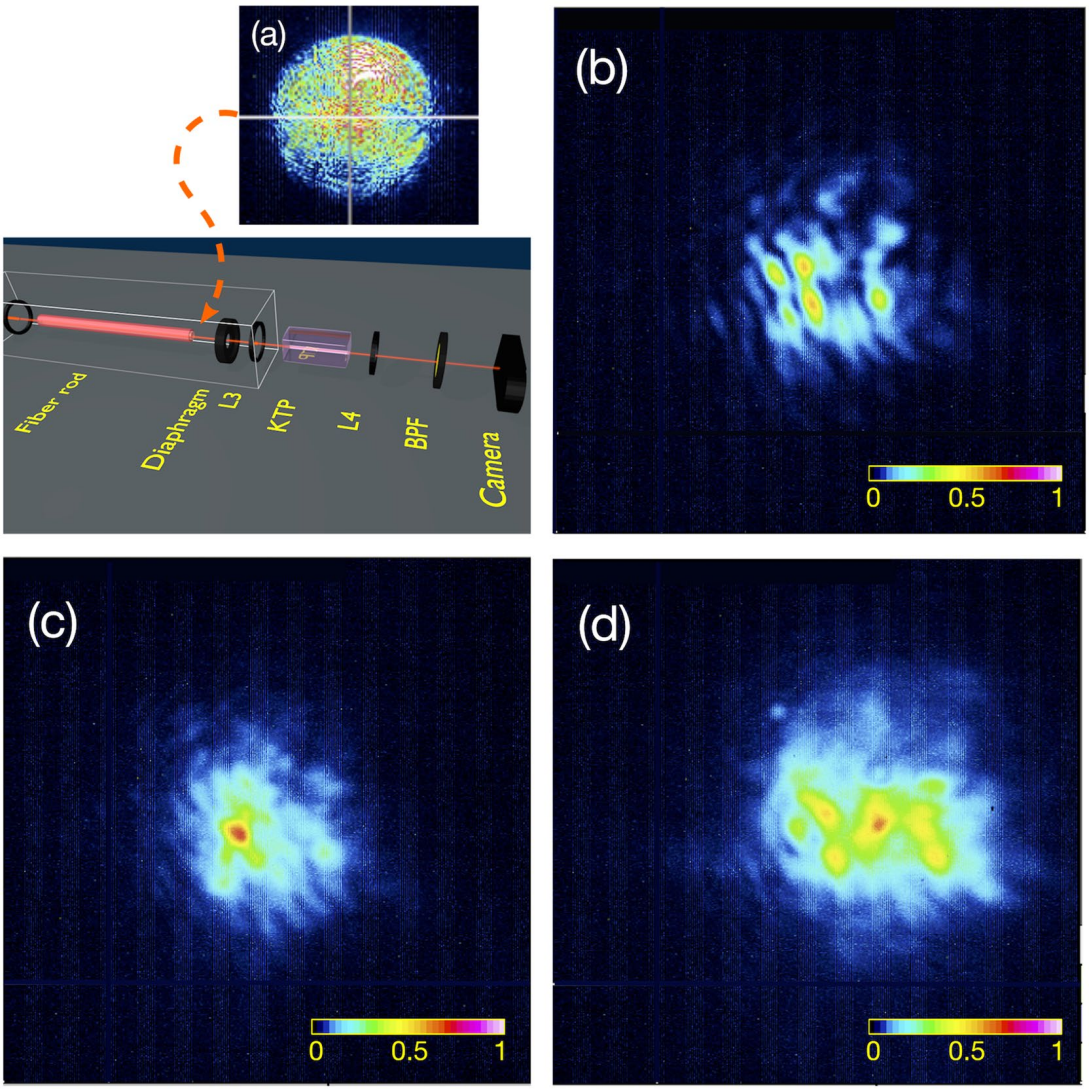
Conditions for quadratic spatial beam self-cleaning. (a) FF beam at the output of the multimode fiber rod for an energy of 0.25 *mJ*; the speckled beam shows that no nonlinear beam reshaping occurs in the MMFR; (b) FF beam at the KTP crystal output for a FF beam energy of 0.06 *μJ* and θ = 1^◦^; FF beam at the KTP crystal output for a FF beam energy of 0.25 *mJ*, for θ = 1^◦^ (c) and θ = 3.5^◦^ (d).

Figure 1 provides a synoptic scheme of our experimental setup for spatial beam cleaning by SHG in a quadratic nonlinear crystal. The nearly Gaussian beam from the laser was spatially scrambled, by propagating it through a short span of highly multimode fiber rod (MMFR) consisting of a 1 mm diameter glass core, surrounded by a polymer cladding. As shown in panel (a) of Fig. 2, at the output of the MMFR we obtained a highly speckled, multimode beam with large numerical aperture. Note that no spatial beam reshaping was observed at the output of the MMFR, even at the highest energy value considered in the present experiments. For effectively coupling this beam into the quadratic KTP crystal, it was necessary to reduce its divergence at the output of the MMFR. For this purpose, we used a diaphragm to filter out the highest spatial frequencies, followed by a lens to collimate the beam into the crystal.

Phase-matching of SHG in the KTP crystal was obtained by exploiting its natural anisotropy. The crystal was cut for type-II SHG, which requires the FF pump power to be equally divided between ordinary and extraordinary axes. All experiments were carried out at room temperature: the degree of mismatch from the phase-matching condition between the FF and the SH could be controlled by tilting the crystal, which changes the incidence angle between the beam and the crystal facet.

At relatively low FF beam energies, that is before any nonlinear spatial beam reshaping occurs, a speckled intensity pattern was observed at the output of the KTP crystal (see Fig. 2(b)). On the other hand, Fig. 2(c) shows that, at sufficiently high FF beam energies (for an input FF beam with angle θ = 1^◦^ and a beam energy of 0.25 *mJ*), the FF beam self-cleans into a single spot, sitting on a low-power background of noncollinear beams. Figure 2(d) shows that, as the SHG gets well out of phase-matching because of crystal rotation, (the crystal angle is rotated θ = 3.5^◦^), the self-cleaning effect disappears: the output beam is again speckled, which denotes that the beam exhibits a highly spatially multimode content.

**Figure 3:**
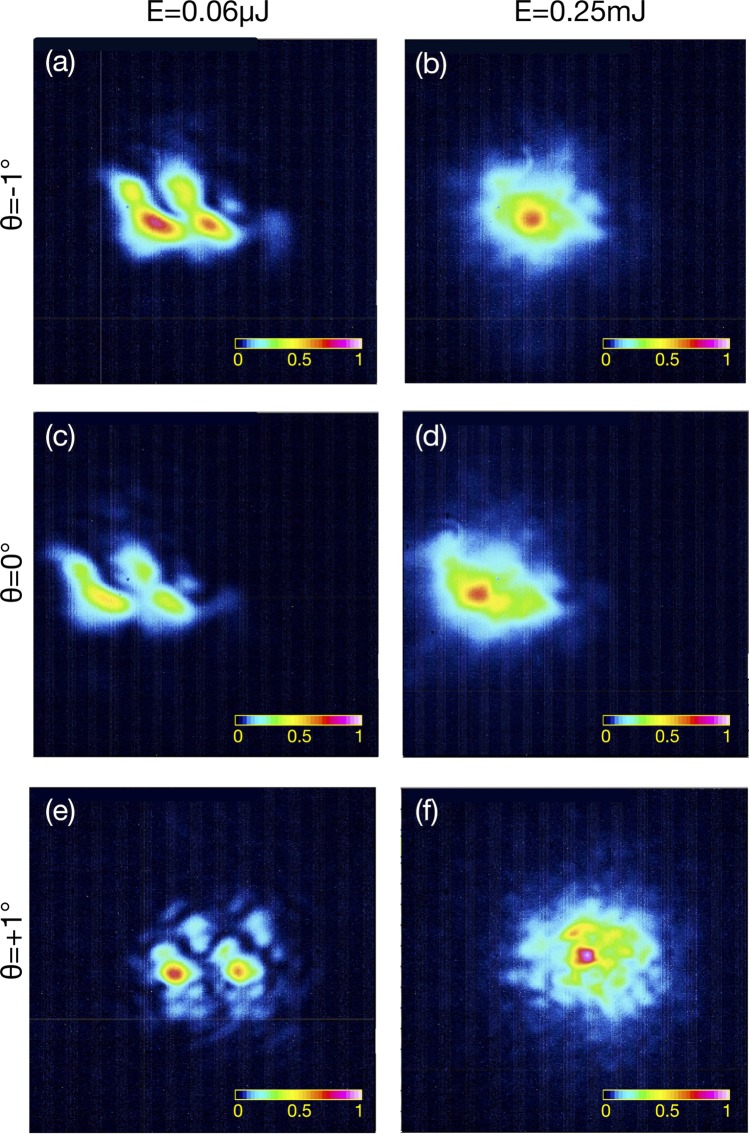
Persistence of spatial beam self-cleaning. FF beam intensity distribution at the output of the KTP crystal and energy 0.06 *μJ* (panels (a,c,e)) and 0.25 *mJ* (panels (b,d,f)). Panels (a,b): input angle θ = −1^◦^; panels (c,d): input angle θ = 0^◦^; panels (e,f): input angle θ = +1^◦^.

In the experimental results reported in Fig. 3 we can see that beam self-cleaning does occur for zero, small positive and even negative crystal angular offsets around the phase-matching condition. Note that the low-energy images in Fig. 3 (panels (a), (c), (e)) show two replicas of the same speckles: this is due to the anisotropy, and consequent spatial walk-off in the KTP crystal. Nevertheless, at high energies (panels (b), (d), (f)) all spots appear to merge into a single beam.

**Figure 4:**
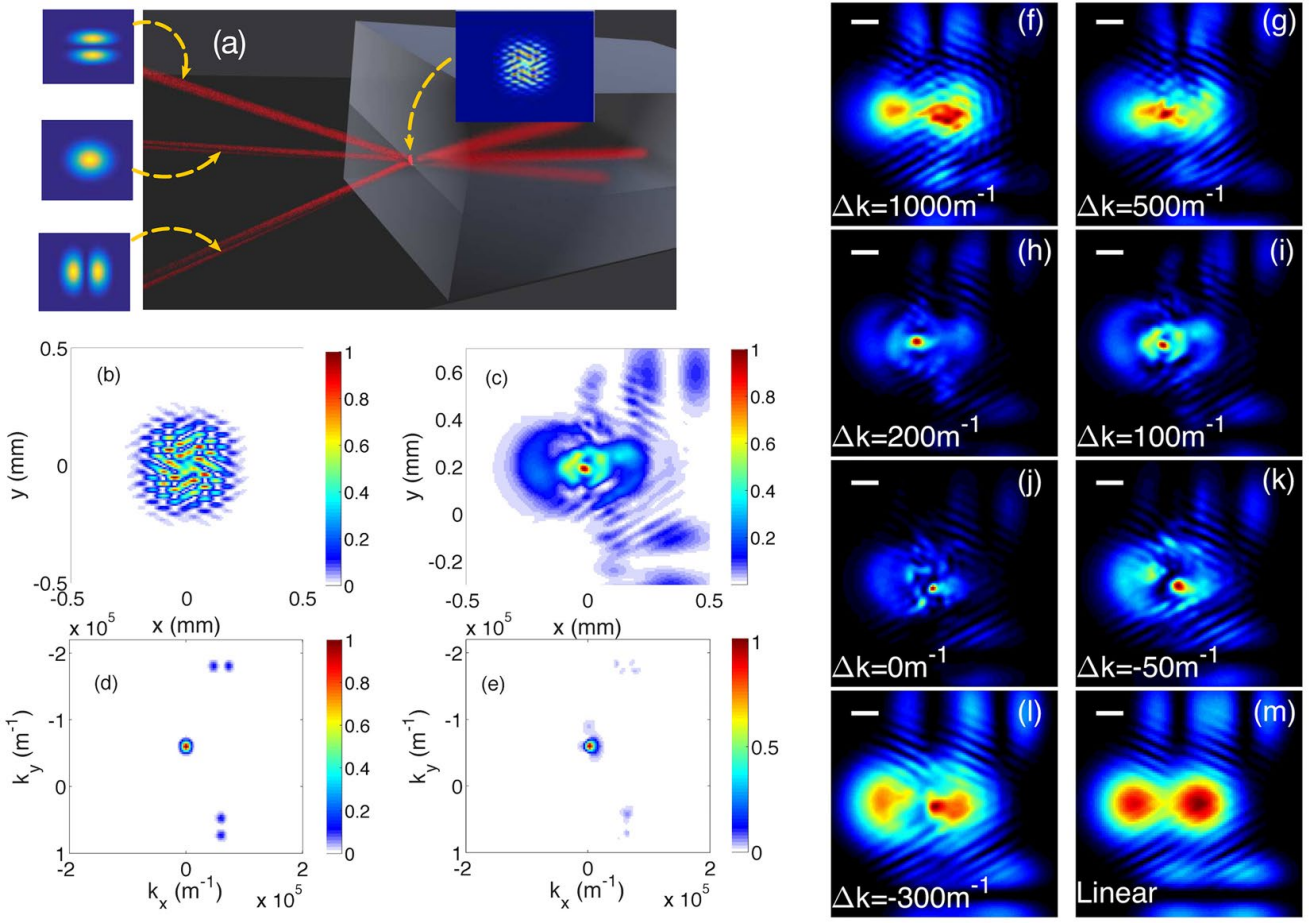
Simulation of spatial beam self-cleaning. (a) schematic illustration of the FF input conditions in the KTP crystal; Bottom left: total intensity distribution at the input (b) and output (c) of a 30-*mm* long KTP crystal at input peak intensity of 1 *GW*/*cm*^2^ and linear phase mismatch ∆*k* = 100 *m*^−1^; the corresponding angular spectrum (d,e) shows substantial spatial frequency conversion into the condensate (i.e., with *k*_*x*_ = *k*_*y*_ = 0) mode; panels (f–m): dependence on linear phase mismatch ∆*k* of numerical FF beam total intensity distribution.

The spatial self-cleaning process of the FF beam, that we observed as a result of SHG, could be qualitatively well reproduced by numerical simulations of type-II SHG. For details about the model equations, see Eq. 1 in the Materials and Methods. In the numerics we considered a simplified model of a multimode input FF beam, consisting in a linear superposition of noncollinear Hermite-Gauss beams with randomly chosen angles and relative phases (see the scheme in Fig. 4(a)). The input intensity was set to be equal to 1 *GW*/*cm*^2^, for a crystal length of 30 *mm*. Moreover, we used the quadratic coefficient *d*_*e f f*_ = 2.5 *pm*/*V*. Figure 4(a) also shows the resulting input intensity, after coherent combining at the crystal input of the multimode beam components.

Panels (b)-(e) of Fig. 4 provide a theoretical support for the observations, based on the full numerical solution of the three-wave equations including beam diffraction, as described in the Materials and Methods. As can be seen, the input multimode beam is reshaped by SHG into a single, self-cleaned beam sitting on a low intensity background. Moreover, Fig. 4 (panels (d) and (e)) shows that, at sufficiently high intensities the SHG process leads to a substantial transfer of energy from high spatial frequencies of the FF beam into the spatial frequency component that propagates on axis (i.e., with *k*_*x*_ = *k*_*y*_ = 0).

The simulation results in the various panels (f)-(m) of Fig. 4 provide a theoretical confirmation of the observed role of the SHG phase mismatch ∆*k* on the control of the spatial FF beam cleaning effect. For reference, the output beam in the linear regime is shown in the last panel (m), again showing the emergence of two replicas of the speckled beam, owing to the crystal anisotropy. In the nonlinear regime, the results are strongly dependent on the value of phase mismatch. For instance, panel (f) closely resembles the linear case (m). When the magnitude of the phase mismatch is gradually reduced (panels (g), (h), (i)), the output beam shape appears strongly reshaped towards a single on-axis beam, sitting on a low-intensity background. The positive (negative) sign of the phase mismatch indicates that the FF experiences a self-focusing (defocusing) effect, at least in the well-known cascading limit. As we will see in the next paragraph, at high FF intensities and close to phase-matching, FF beam self-focusing occurs for both signs of the phase-mismatch.

Simulations show that, once created, besides some fluctuations in its diameter, the reshaped beam stably maintains its shape upon subsequent propagation. In agreement with experiments, simulations also reveal the emergence of a single spot at the crystal output for zero (Fig. 4(j)) or negative mismatch values (Fig. 4(k)). On the other hand, for a relatively large negative mismatch, the FF beam self-cleaning is no longer sustained (Fig. 4(l)). With small mismatch values, light localization is mainly controlled by the beam energy, for both signs of the linear wave vector mismatch.

In this work we focus our attention to the nonlinear beam cleaning of the FF beam: interestingly, our simulations (not shown here) reveal that FF beam cleanup is accompanied by a similar spatial cleaning of the SH beam, which also emerges as a collinear beam generating a single bright spot at the crystal output.

### Nonlinear conversion bandwidth

In a second series of experiments, aimed at unveiling the mechanism leading to spatial beam self-cleaning, we characterized the nonlinear response of the quadratic KTP crystal in the spatial and in the angular frequency domain, around the phase-matching condition. In order to do that, this second series of experiments was carried out in absence of the MMFR for input beam scrambling (white box of Fig. 1). The beam was linearly polarized by a cube polarizer, and subsequently the orientation of the state of polarization (SOP) was controlled by a half-wave plate, so that the linear SOP was set at 45^◦^ from the ordinary and extraordinary axes.

The FF input beam diameter was chosen so that the associated diffraction length *L*_*D*_ was approximately equal to the physical length of the crystal L. Under these conditions, one may neglect the effect of diffraction when such a wide laser beam is directly coupled into the crystal: the conversion efficiency is only ruled by the magnitude of the nonlinearity, the FF intensity, and the phase-matching or incidence angle. Even under these conditions, our experiments revealed that near phase-matching the SHG process may strongly modify the spatial beam shape. Note that the direct use of the laser beam, with much better quality and significantly lower divergence than the beam at the output of the MMFR that, strongly reduces the energy required to observe spatial or transverse nonlinear effects in the KTP crystal.

**Figure 5:**
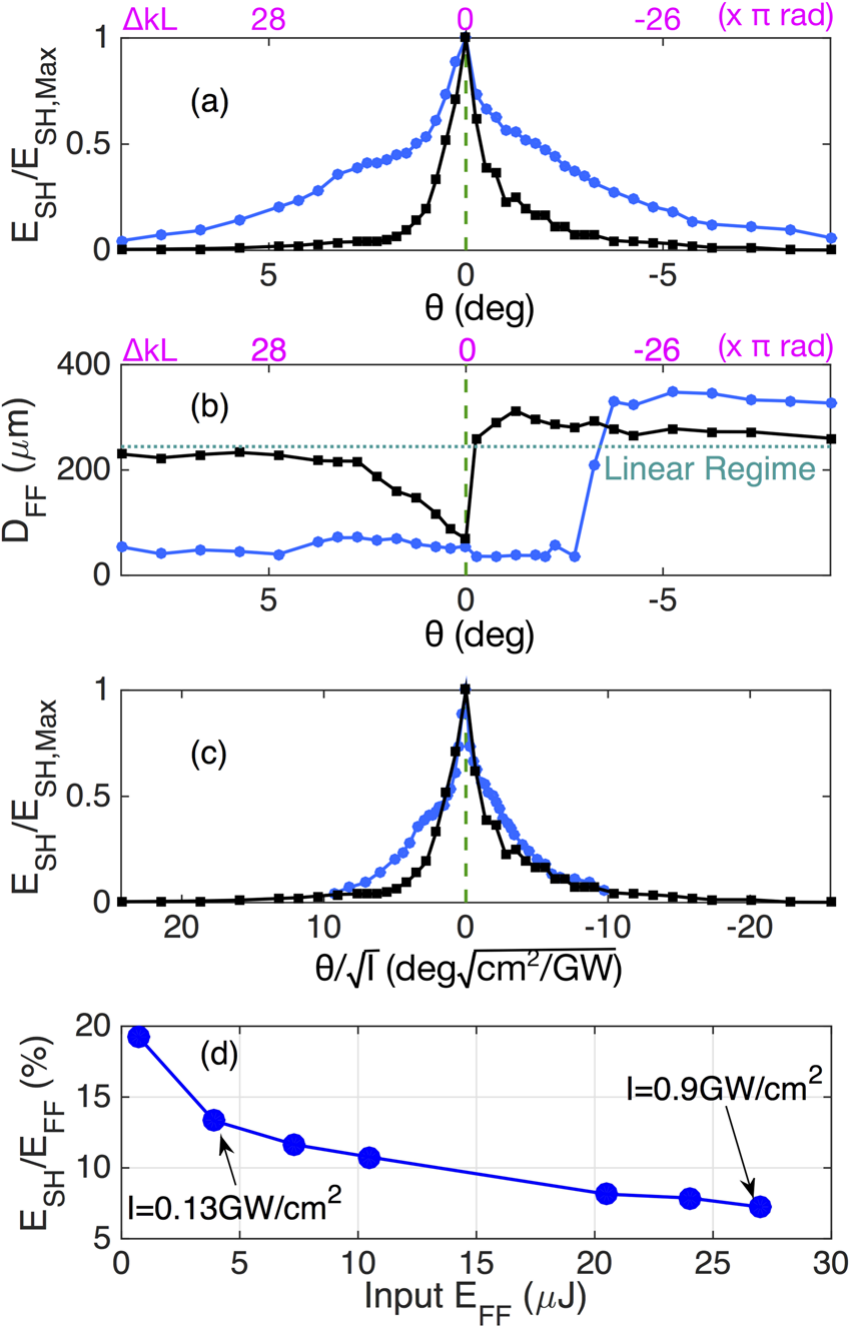
Beam self-compression. Relative efficiency of SHG upon phase mismatch at low input intensity (black curve 0.13 *GW*/*cm*^2^) and at high intensity (blue curve 0.9 *GW*/*cm*^2^) in KTP crystal. Panel (a) shows the result with respect to the input angle θ. Panel (b) shows the corresponding diameter at FF. Panel (c) shows the same results of panel (a) upon an abscissa proportional to κ. Input beam diameter 250 *μm*. Panel (d): experimental results of SHG efficiency vs. input beam energy, at the phase-matching point θ = 0^◦^; here arrows indicate the two values of input FF intensity corresponding to the black squares and blue dots of curves in panels (a–c).

In Fig. 5 we illustrate the dependence of SHG relative (with respect to the phase-matched value) efficiency upon KTP orientation angle θ. As can be seen, at high energy levels the nonlinear conversion (or acceptance) bandwidth of SHG grows larger, in agreement with the nonlinear theory of SHG^37,38^. In fact, Fig. 5(c) shows that the conversion bandwidth remains nearly constant, when the physical angle (approximately proportional to linear wave vector mismatch) is normalized by the square root of the pump intensity. Since diffraction can be neglected, at least in the early stages of propagation, as discussed in the Supplementary Materials, the model of Refs. ^39,40^ can explain well the observed nonlinear rescaling of the SHG efficiency bandwidth. This purely nonlinear effect has an equivalent counterpart for cubic nonlinearities, as discussed in ref. ^41^.

To evaluate the actual value of the SHG efficiency in our experiments, we measured the output SH beam energy vs. input FF beam energy at the phase-matching point θ = 0. The resulting dependence on FF beam energy of the SHG efficiency, calculated as the ratio of SH and FF beam energies, is illustrated in panel (d) of Fig. 5. In this figure, arrows indicate the two values of input FF intensity corresponding to the black squares and blue dots of curves in Fig. 5(a–c). As can be seen, because of the nonlinear saturation of the SHG, owing to the combined effects of self-focusing, FF spectral broadening and crystal walk-offs^38^, its efficiency decreases from the low pump beam intensity value of 20% down to below 8% for pump energies above 25 *μJ*.

### Spatial beam compression

Most interestingly, our experiments also reveal that at high intensities there is a spatial compression of the FF beam, for both positive and negative mismatch values. In fact, Fig. 5(b) shows that the beam defocusing (or spatial broadening) which is observed for small negatives input angles (from 0° to −3^◦^) at low intensity 0.13 *GW/cm*^2^, is transformed into a beam self-focusing (or spatial compression) when the FF intensity is increased up to 0.9 *GW*/*cm*^2^ (see Fig. 5(b)). Figure 5(b) shows that, for θ > −3^◦^, the output beam diameter shrinks from 250 *μm* down to about 50 *μm* with an input intensity of 0.9 *GW*/*cm*^2^.

## Discussion

We have experimentally demonstrated a novel property of second-harmonic generation in quadratic nonlinear media. Namely, that close to phase-matching with the generated second-harmonic, a sufficiently intense, initially highly multimode optical beam self-increases its brightness. In other words, the output pump beam evolves from a speckled intensity pattern into a cleaned beam propagating along the crystal axis. Experimental observations are confirmed by numerical simulations across the phase-matching bandwidth by using a general model for three-wave mixing, including spatial walk-off and diffraction.

To study the mechanism of quadratic beam cleaning, we experimentally characterized the nonlinear spatial response of the crystal by using a quasi-plane wave beam. At relatively high FF beam intensities, a significant self-focusing of the FF beam is observed around the phase-matching condition, for both negative and positive wave vector mismatches. As mentioned in the Introduction, close to phase-matching the nonlinear contribution to the propagation of the FF beam can be approximated by a third-order or cubic non-local nonlinear response. However, because a beam comprising a large number of spatial wave vectors is self-cleaned into a single collinear beam, the observed beam self-cleaning effect based on propagation in a quadratic nonlinear medium appears of a stronger nature than Kerr beam self-cleaning, which conversely requires that a predominant excitation of the fundamental beam is present at the input plane.

The observed nonlinear spatial beam reshaping and clean-up has the potential to be exploited across a variety of laser technologies, ranging from parametric frequency conversion sources to high-harmonic generation.

## Materials and Methods

### Experimental set-up

In our experiments, we used a high-energy Q-switched mode-locked Nd:YAG laser with 20 Hz repetition rate emitting 30 ps pulses at 1064 nm. In the beam cleaning experiments, the laser beam was spatially scrambled by a 10-cm long MMF with a 1 *mm* glass core diameter, surrounded by a polymer cladding. Before coupling into the MMF, the laser beam was collimated to a 900 *μm* diameter by two lenses with f = 100 *mm* (L1 in Fig. 1) and f = −30 *mm* focal length (L2 in Fig. 1), respectively. To reduce the divergence of the beam at the output of the MMF, we used a diaphragm to filter out the highest spatial frequencies, and a lens of f = 75 *mm* (L3 in Fig. 1) to collimate the remaining beam into a KTP crystal. We used a 30-mm long sample of type II KTP. All experiments were carried out at room temperature, and the phase mismatch was varied by tilting the crystal, in order to change the incidence angle between the beam and the crystal facet. The beam was linearly polarized by a cube polarizer, and subsequently the SOP orientation was controlled by a half-wave plate to meet the requirement of type II SHG. The beam size was limited by one diaphragm. We experimentally analyzed the energy of the generated SH wave with a system composed of a band-pass filter and an energy meter. In addition, we used a CCD camera to measure the spatial shape of the output FF beam.

### General model for three-wave mixing

Let us recall here the model commonly used to describe beam propagation in diffractive bulk quadratic media, and used in our numerical simulations. Limiting the discussion to type-II SHG, under the assumption of slowly varying envelopes, and neglecting temporal effects, one obtains the three nonlinearly coupled equations:

1 $$\begin{array}{c}\left(\frac{\partial }{\partial z}-{\rho }_{0}\frac{\partial }{\partial x}\right){E}_{0}+\frac{1}{2i{k}_{0}}{\nabla }_{\perp }^{2}{E}_{0}=i{\chi }_{0}^{(2)}{E}_{1}^{\ast }{E}_{2}{e}^{-i\Delta kz}\\ \left(\frac{\partial }{\partial z}-{\rho }_{1}\frac{\partial }{\partial x}\right){E}_{1}+\frac{1}{2i{k}_{1}}{\nabla }_{\perp }^{2}{E}_{1}=i{\chi }_{1}^{(2)}{E}_{0}^{\ast }{E}_{2}{e}^{-i\Delta kz}\\ \left(\frac{\partial }{\partial z}-{\rho }_{2}\frac{\partial }{\partial x}\right){E}_{2}+\frac{1}{2i{k}_{2}}{\nabla }_{\perp }^{2}{E}_{2}=i{\chi }_{2}^{(2)}{E}_{0}^{\ast }{E}_{1}{e}^{-i\Delta kz}\end{array}$$

where *E*_*j*_ is the envelope of the electric field, considered as a quasi-plane monochromatic wave, Δ*k* = *k*_0_ + *k*_1_ − *k*_2_ is the phase mismatch, with |∆*k*| ≪ *k*_*j*_. Coefficients *ρ*_*j*_ account for the energy walk-off related to crystal anisotropy, ${\chi }_{j}^{(2)}={\omega }_{j}{d}_{eff}/(n({\omega }_{j})c),$ where *d*_*eff*_ is the relevant nonlinear quadratic coefficient. Moreover, *k*_*j*_ = *n*_*j*_ ω_*j*_/*c* is the free-space wave vector for wave *j* and ω_0_ = ω_1_ + ω_2_/2. The model described by Eq. 1 is particularly well suited to study spatial solitons, where diffraction competes with the quadratic nonlinear effect. In the cascading limit, one observes beam self-focusing for ∆*k* > 0, and beam self-defocusing for ∆*k* < 0.

## Supplementary information

Supplementary Information.
