# Supplementary material for: Spatial Beam Self-Cleaning in Second-Harmonic Generation
Source: Sci Rep. 2020 Apr 29;10:7204. doi: 10.1038/s41598-020-64080-7 (PMC7190728; doi:10.1038/s41598-020-64080-7)
Supplement: Supplementary file 1 — Supplementary Information. [file 41598_2020_64080_MOESM1_ESM.pdf]

Supplementary Materials for

Spatial Beam Self-Cleaning in Second-Harmonic Generation

K.Krupa,[1,2*] R. Fona,[3,9] A. Tonello,[4] A. Labruyère,[4] B. M. Shalaby,[4,8] S. Wabnitz,[5,6] F. Baronio,[3] A.B. Aceves,[7] G. Millot,[2] V. Couderc[4]

[1]Institute of Physical Chemistry of the Polish Academy of Sciences
ul. Kasprzaka 44/52, 01-224 Warsaw, Poland
[2]Université Bourgogne Franche-Comté, ICB
UMR CNRS 6303, 9 Av. A. Savary, 21078 Dijon, France
[3]Dipartimento di Ingegneria dell'Informazione, Università di Brescia
Via Branze 38, 25123 Brescia, Italy
[4]Université de Limoges, XLIM, UMR CNRS 7252
123 Av. A. Thomas, 87060 Limoges, France
[5] DIET, Sapienza University of Rome
Via Eudossiana 18, 00184 Rome, Italy
[6]Novosibirsk State University
Pirogova 1, Novosibirsk 630090, Russia
[7]Department of Mathematics, Southern Methodist University
Dallas, Texas 75275-0156, USA
[8]Physics Department, Faculty of Science, Tanta University
Tanta, Egypt
[9]Zerogroup, Brescia, Italy

*To whom correspondence should be addressed; E-mail: kkrupa@ichf.edu.pl.

March 19, 2020

Nonlocal cubic nonlinear equation for the FF beam Assuming for simplicity that, in Eq. (1) of the Materials and Methods section of the main text, $E_0 = E_1$, $\rho_1 = 0$, and neglecting the y dependence, one obtains the coupled equations

$$\frac{\partial E_1}{\partial z} + \frac{1}{2ik_1}\frac{\partial^2 E_1}{\partial x^2} = i\kappa E_1^* E_2 e^{-i\Delta kz}, \tag{1}$$

$$\frac{\partial E_2}{\partial z} - \rho_2 \frac{\partial E_2}{\partial x} + \frac{1}{2ik_2}\frac{\partial^2 E_2}{\partial x^2} = i\kappa E_1^2 e^{i\Delta kz}, \tag{2}$$

where the nonlinear coupling strength is described by κ, normalised such that $|E_1|^2$ and $|E_2|^2$ are now measured in Watts.

It is possible to obtain a single equation for the FF beam as follows [1] Eq. (2) is first solved formally in the Fourier domain, to obtain

$$\mathscr{F}\left[E_2(z,x)\right] = i\kappa e^{\hat{k}z}\int_0^z e^{(i\Delta k-\hat{k})z'}\mathscr{F}[E_1^2(z',x)]\,dz' \tag{3}$$

where we used $E_2(0,x) = 0$, $\mathscr{F}\left[\cdot\right] = \int_{-\infty}^{\infty} \cdot\, e^{ik_x x'}\,\mathrm{d}x'$ denotes Fourier transformation, and $\hat{k}(k_x) = i\left[-\rho_2 k_x + (1/2k_2)k_x^2\right]$. We then substitute $E_2(z,x)$ into Eq. (1), which leads to

$$i\frac{\partial E_1}{\partial z} + \frac{1}{2k_1}\frac{\partial^2 E_1}{\partial x^2} = -i\kappa^2 E_1^* \mathscr{F}^{-1}\left[\int_0^z e^{(\hat{k}-i\Delta k)(z-z')}\mathscr{F}[E_1^2(z',x)]\,dz'\right]. \tag{4}$$

Eq. 4 can be simplified if the FF is assumed to evolve slowly enough over a crystal length L, so that to first order its z-variation can be neglected when computing the integral. In this case one may average the right-hand side of Eq. (4) over the length L, and obtain the single evolution equation for the FF beam

$$i\frac{\partial E_1}{\partial z} + \frac{1}{2k_1}\frac{\partial^2 E_1}{\partial x^2} + i\sigma E_1^*[E_1^2(z,x)\otimes I(x)] = 0, \tag{5}$$

where $\sigma = \kappa^2 L$, $\otimes$ denotes convolution, and $I(x) = \mathscr{F}^{-1}[\hat{I}(k_x)]$ is a response function given by

$$\hat{I}(k_x) = \frac{1}{L^2}\int_0^L\int_0^z e^{(\hat{k}-i\Delta k)(z-z')}\,dz'dz = (1/2)\mathrm{sinc}^2(s/2) + i(1/s)(\mathrm{sinc}(s)-1), \tag{6}$$

with $s(k_x) = [\Delta k + i\hat{k}(k_x)]L$. According to Eq. (5), the FF beam experiences an effective nonlocal third-order nonlinearity. Note the phase dependence of the nonlocal response, since the square of the field, rather than the intensity, is involved. The real part of the response function represents a spatial frequency dependent nonlinear loss, while the imaginary part represents phase modulation. Moreover, it can be shown that the spatial domain response $I(x)$ vanishes outside of $0 \leq x \leq -\rho_2 L$ for $\rho_2 < 0$ (or $-\rho_2 L \leq x \leq 0$ for $\rho_2 > 0$). Thus spatial walk-off determines the extent of the nonlocal response. The SH field is slaved to the FF, see Eq. (3), so that the output field at the SH reads as

$$E_2(L,x) = i\kappa \mathscr{F}^{-1}\left[\frac{e^{\hat{k}L} - e^{i\Delta kL}}{\hat{k} - i\Delta k}\mathscr{F}[E_1^2]\right]. \tag{7}$$

Asymmetric nonlinear conversion efficiency and spatial beam narrowing in PPLN. To investigate the generality of nonlinear beam reshaping in SHG, we also carried out experiments of SHG conversion efficiency and spatial beam reshaping as a function of the linear phase mismatch by using quasi-phase-matching (QPM) in a periodically poled Lithium Niobate (PPLN) crystal. In this case, the transverse electric field of the beam was oriented along the extraordinary axis of the crystal. We used a 15 mm long sample of PPLN, and we varied the linear phase mismatch (Δk) by simply changing the crystal temperature.

A summary of our results is shown in Fig.S1. Panel (a) shows the relative efficiency of SHG upon mismatch. As can be seen in Fig.S1(a), as the FF power grows larger (blue curve), the broadening of the SHG conversion efficiency is strongly asymmetric on opposite sides of the phase-matching condition (vertical dashed line). The degree of such asymmetry is larger in the self focusing region (i.e., with $\theta > 0$). On the other hand, for moderate pump power (black curve), the SHG conversion curve remains nearly symmetrical. In Fig.S1(b) we illustrate the corresponding diameter values for the two cases of moderate (black) and high (blue) pump powers. These measurements that also spatial beam reshaping is subject to an asymmetric

dependence on the sign of the linear phase mismatch. In Fig.S1, the horizontal dashed line indicates the output beam diameter in the linear regime.

The presence of such asymmetric SHG efficiency can be qualitatively well reproduced by a simple coupled wave model (Eqs. (11)). In fact, since diffraction can be neglected in these expriments, at least in the early stages of propagation, the model of Refs.[2, 3] can explain the main observed features. Note that the build up of a progressive asymmetry in SHG efficiency between positive and negative angles, albeit with a reduced magnitude, is also observed in the KTP experiments of Fig.5. Full numerical simulations (here not shown) including both diffraction and nonlinearity confirm quantitatively well the observed asymmetry (see Ref.[4]).

Coupled wave reduced model of SHG. When the input beams are relatively wide, so that the crystal length is smaller than the Rayleigh distance, it is possible to simplify Eqs. (1), reported in section Material and Methods of the main text, by neglecting derivatives with respect to the transverse dimensions. Care should be taken to adopt this approximation for those cases where the nonlinear beam reshaping, which occurs along the crystal, drastically changes the beam waveform (i.e. nonlinear self-focusing). Close to phase matching, it is possible to describe the main features of the nonlinear interaction between a FF wave, at angular frequency ω_0 and its SH wave, at $2\omega_0$, in terms of simple integrable nonlinear dynamical system or equivalent particle nonlinear oscillator. In the limit of plane waves and the continuous wave regime (supposing that diffraction, walk-off and dispersion can be neglected), by clever use of conserved quantities, one arrives to a global picture based on a phase plane analysis [2, 3].

Starting from following system of equations

$$\frac{\partial E_0}{\partial z} = i\chi_0^{(2)} E_0^* E_2 e^{-i\Delta kz}$$

$$\frac{\partial E_2}{\partial z} = i\chi_2^{(2)} \frac{1}{2} E_0^2 e^{+i\Delta kz} \tag{8}$$

and following a standard procedure, one can normalize the envelope $E_j = \mathscr{A}_j\sqrt{2/(c\varepsilon_0 n(\omega_j))}$, and rewrite Eqs. (8) as

$$\frac{d\mathscr{A}_0}{dz} = i\omega_0 R \mathscr{A}_0^* \mathscr{A}_2 e^{-i\Delta kz}$$

$$\frac{d\mathscr{A}_2}{dz} = i\omega_0 R \mathscr{A}_0^2 e^{+i\Delta kz} \tag{9}$$

with $R = d_{eff}\sqrt{2}/(cn(\omega_0)\sqrt{c\varepsilon_0 n(\omega_2)})$.

One immediate outcome of the theory is the prediction of a symmetric behavior of the conversion efficiency around exact phase matching. On the other hand, our experimental results (see in particular Fig.S1(a)) show the presence of a clear asymmetry of the SHG efficiency. Such asymmetry can be related to the nonlinear transverse beam reshaping (i.e., beam compression or broadening) which occurs opposite sides of the phase-matching condition, as shown in Fig.S1(b). In order to take into account of such nonlinear beam reshaping, it is necessary to modify the plane-wave model of Refs [2, 3], by projecting the evolution equations into their respective transverse modes. With this in mind, if $\mathscr{A}_0 = A_0(z)F_0(x,y), \mathscr{A}_2 = A_2(z)F_2(x,y)$, one

may describe the coupling among the FF and SH waves in terms of the equations

$$i\frac{dA_0}{dz} = -\omega_0 R \frac{C_{02}}{C_0} A_0^* A_2 e^{-i\Delta k z}$$

$$i\frac{dA_2}{dz} = -\omega_0 R \frac{C_{02}}{C_2} A_0^2 e^{+i\Delta k z} \tag{10}$$

where $C_{02} = \iint F_0^2(\underline{x}) F_2(\underline{x}) d\underline{x}$, $C_h = \iint F_h^2(\underline{x}) d\underline{x}$, account for the effective transverse effects ($h = 0, 2$). These coefficients depend on the linear phase mismatch Δk, thus breaking the symmetry when its sign is changed. The envelope A_0 (FF) and A_2 (SH) are taken proportional to the electric field, in a way that $|A_j|^2$ gives directly the beam power at wave j. Normalizing the amplitudes by the use of the conserved quantity $P = C_0|A_0|^2 + C_2|A_2|^2$ so that $a_0 = A_0\sqrt{2/P}$, $a_2 = A_2 \exp(-i\Delta k z)\sqrt{C_2/(C_0 P)}$ and rescaling $Z = \rho z$, $\rho(\Delta k) = \omega_0 R C_{02}\sqrt{P/(C_0 C_2)}$, Eqs. (10) recover Eqs. (2-4) in Ref.[3], and the corresponding phase portrait.

$$i\frac{da_0}{dZ} = -a_0^* a_2$$
$$i\frac{da_2}{dz} = -\kappa a_2 - \frac{1}{2} a_0^2 \tag{11}$$

In this reduced dynamical system, $\kappa = -\Delta k/\rho(\Delta k)$ is a function of P and of the linear mismatch Δk. As already pointed out in [3], κ plays the role of a power dependent mismatch parameter: when the input power P is increased, one needs to increase the linear mismatch Δk in order to keep the same value for κ. This improved theory then predicts a broadening of the conversion efficiency of SHG upon linear mismatch Δk. One can notice that the current expression for κ not only accounts for the effect of the transverse profiles, but it also involves an asymmetric outcome near $\Delta k = 0$, since in general $\kappa(\Delta k) \neq \kappa(-\Delta k)$ for $|\Delta k| << 1$. In particular, in the presence of spatial beam self focusing, the ratio $C_{02}/\sqrt{C_0 C_2}$ may increase, thus enhancing the nonlinear conversion. The opposite situation may occur in the presence of spatial beam

defocusing along the propagation in the crystal, because the same parameters will reduce the nonlinear conversion. A detailed comparison between theory, extensive numerical simulations and experimental output will be left for future work; here we simply wanted to highlight that the present improved model now accounts for the asymmetries that are seen in the spatial profiles in the vicinity of perfect phase matching.

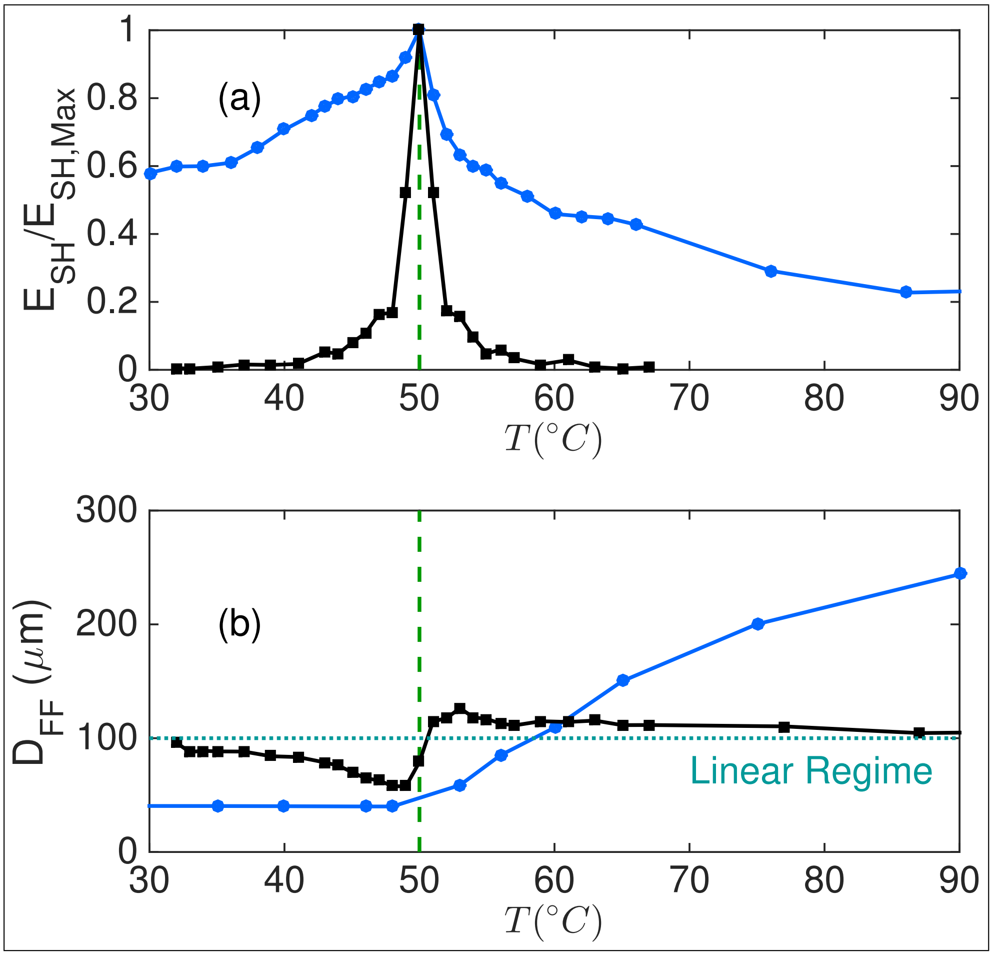

Figure S1: **Asymmetric spectral acceptance and beam self-compression.** Experimental relative efficiency and beam compression for SHG as a function of PPLN crystal temperature. The input pump intensity was 15 MW/cm^2 (black curve) and 5.5 GW/cm^2 (blue curve). Input beam diameter 100 μm. Self-focusing is observed for temperatures below 50 $°C$. Panel (**a**) shows SHG efficiency upon the crystal temperature, panel (**b**) the corresponding beam diameter.

References

[1] Leo, F. *et al.* Walk-Off-Induced Modulation Instability, Temporal Pattern Formation, and Frequency Comb Generation in Cavity-Enhanced Second-Harmonic Generation. *Phys. Rev. Lett.* **116**, 033901 (2016).

[2] Trillo, S. & Wabnitz, S. Nonlinear parametric mixing instabilities induced by self-phase and cross-phase modulation. *Opt. Lett.* **17**, 1572–1574 (1992).

[3] Trillo, S., Cappellini, G., Wabnitz, S. & Chisari, R. Two-wave mixing in a quadratic nonlinear medium: bifurcations, spatial instabilities, and chaos. *Opt. Lett.* **17**, 637–639 (1992).

[4] Krupa, K. *et al.* Self-increased acceptance bandwidth of second harmonic generation for high-energy light sources. In *2015 Spatiotemporal Complexity in Nonlinear Optics (SCNO)*, 1–3 (2015).
